# Senescence dynamics define therapeutic windows for Duchenne muscular dystrophy in DBA/2-mdx mice

**DOI:** 10.1186/s13395-026-00426-5

**Published:** 2026-05-02

**Authors:** Aina Calls-Cobos, Aida Beà Tàrrega, Andrés Cisneros, Megan Rommelfanger, Silvia Campanario, Mercedes Grima-Terrén, Ignacio Ramírez-Pardo, Victoria Moiseeva, Vera Lukesova, Eva Andrés, Grace Chou, Yuewen Zheng, Nasun Hah, Albert Blasco-Roset, Ana Planavila, Carolina Soler-Botija, Antoni Bayés-Genís, Eusebio Perdiguero, Antonio L. Serrano, Pura Muñoz-Cánoves

**Affiliations:** 1 Altos Labs, San Diego Institue of Science, San Diego, USA; 2 Department of Medicine and Life Sciences, Universitat Pompeu Fabra (UPF), Barcelona 08003, Spain; 3 Departament de Bioquímica i Biologia Molecular, Institut de Biomedicina (IBUB), Universitat de Barcelona and CIBER Fisiopatología de la Obesidad y Nutrición, Barcelona, 08028 Spain; 4 ICREC Research Lab, Germans Trias i Pujol Research Institute (IGTP), Barcelona, Spain; 5 CIBER Cardiovascular, Instituto de Salud Carlos III, Madrid, Spain; 6 Heart Institute (iCOR), Germans Trias i Pujol University Hospital, Badalona, Barcelona, Spain; 7 Department of Medicine, Autonomous University of Barcelona, Barcelona, Spain

**Keywords:** Cardiac remodeling, Cardiomyopathy, Cellular senescence, Dasatinib and quercetin, Duchenne muscular dystrophy, Fibrosis, Mouse models, Preclinical research, Senolytic therapy, Skeletal muscle regeneration, Therapeutic timing

## Abstract

**Background:**

Duchenne muscular dystrophy (DMD) is a severe X-linked disorder marked by progressive muscle degeneration and regeneration, inflammation and fibrosis. Cellular senescence has emerged as a potential driver of chronic muscle damage, yet its temporal dynamics and therapeutic relevance remain unclear.

**Methods:**

We analyzed senescent cell burden in skeletal and cardiac muscles of the DBA/2-mdx mouse model, which closely mimics features of human DMD. The senolytic combination of dasatinib and quercetin (D + Q) was administered during early or late disease phases to evaluate the impact of senescent cell clearance. Skeletal muscle strength was measured by grip strength and ex vivo force assays, while cardiac function was assessed by echocardiography. Fibrosis and senescence markers were quantified histologically, and transcriptional changes associated with senolysis were identified using bulk RNA sequencing (RNA-seq).

**Results:**

In skeletal muscle, senescent cells appear and peak during early stages of disease progression (3–5 months), coinciding with high degeneration and regeneration activity, and then decline with age as fibrosis increases. In contrast, in the heart, senescent cells emerge at late stages of disease progression (around 12 months), correlating with heart fibrogenesis. Notably, senolytic intervention in the DBA/2-mdx mice promotes a regenerative and antifibrotic gene signature in both tissues. However, the timing of senolytic therapy determines its efficacy: early treatment with D + Q reduces senescent cell burden, decreases fibrosis, and improves fiber size and contractile performance in skeletal muscle, while later treatment reduces cardiac senescence and fibrosis but does not improve skeletal muscle pathology.

**Conclusions:**

Cellular senescence is a dynamic and targetable feature in DMD, with tissue- and age-specific patterns. It represents a potential modifiable therapeutic target, and temporally optimized senolytic strategies could serve as effective adjuncts to current and emerging DMD treatments.

**Supplementary Information:**

The online version contains supplementary material available at 10.1186/s13395-026-00426-5.

## Background

Duchenne muscular dystrophy (DMD) is a fatal X-linked recessive disorder characterized by progressive loss of muscle mass and function. It is caused by mutations in the dystrophin gene, resulting in the absence of functional dystrophin protein [1]. Due to its critical role in maintaining myofiber stability, the loss of dystrophin leads to muscle fiber fragility and continuous cycles of degeneration and regeneration, accompanied by chronic inflammation and fibrotic tissue deposition that severely exacerbate disease progression.

Skeletal muscle exhibits a robust regenerative capacity. Upon injury, muscle stem cells (MuSCs) activate, proliferate and differentiate into new myofibers or self-renew to maintain the stem cell pool [2]. Loss of dystrophin in MuSCs contributes to the impaired muscle regenerative capacity and hence to muscle wasting [3]. MuSC functions are highly regulated by signals from the surrounding MuSC niche [2], which comprises myofibers and diverse mononucleated cell populations, including immune, endothelial, perivascular and fibro/adipogenic progenitor (FAP) cells [4]. Growing evidence now highlights senescent cells as critical components in this niche, where they directly influence the outcome of muscle regeneration [5–8].

Cellular senescence is a stress-induced state of stable, irreversible cell cycle arrest characterized by the accumulation of cyclin-dependent kinase inhibitors (particularly p16^Ink4a^ and p21^Cip1^). No single universal marker defines senescence, although senescence-associated β-galactosidase (SA-β-gal) activity is commonly used for detection. Senescent cells also develop a senescence-associated secretory phenotype (SASP), a complex secretome that includes pro-inflammatory cytokines and chemokines (e.g., IL6 and Ccl2). Other hallmarks of senescence include DNA damage, telomeric dysfunction, loss of nuclear lamin B1 (Lmnb1) and increased oxidative stress. Senescence plays context-dependent roles, exhibiting both beneficial (e.g., wound healing) and detrimental (e.g., chronic disease) effects in different tissues [9].

In skeletal muscle, partial clearance of senescent cells—using either senolytic drugs or genetic approaches—enhances regeneration following acute injury in both young and aged mice [5, 8]. Similarly, senescent cell ablation in dystrophic rodent models improves muscle function, preserves muscle strength and maintains body weight [10, 11].

DMD cardiomyopathy is characterized by progressive myocardial fibrosis and eventual heart failure [1, 12, 13]. Life expectancy of individuals with DMD typically ranges between 20 and 40 years of age, with death most often resulting from cardiac and/or respiratory failure [1, 14]. Therefore, slowing the progression of myocardial damage is a crucial therapeutic goal for delaying the onset of heart failure. While senescent cells have been detected in the cardiac tissue of canine and murine DMD models [15] their pathophysiological role remains largely unexplored.

Here, we show that in the DBA/2-mdx mouse model of DMD, the temporal dynamics of senescent cell accumulation differ markedly between skeletal and cardiac muscles. Accordingly, optimal therapeutic benefits require interventions that target senescent cells in a timely manner to coincide with their peak abundance in each tissue. Our findings identify senolytic therapies as a promising strategy to slow systemic disease progression in dystrophic striated muscles and support its use as a critical complementary approach to gene therapies addressing the underlying genetic defect.

## Methods

### Mouse models

The mouse lines p16-3MR (kindly donated by J. Campisi [16]), dystrophic DBA/2-mdx and DBA/2-mdx^p16−3MR^ (dystrophic DBA/2-mdx mice crossed with p16-3MR mice) were bred and aged at the animal facility of the Barcelona Biomedical Research Park (PRBB). DBA/2 mice were used as control wild type (WT) mice. Animals were housed in standard cages under a 12-hour light/dark cycle and fed ad libitum with a standard chow diet. All experimental protocols were approved by the Catalan Government, following applicable legislation and the guidelines of the PRBB Institutional Animal Care and Use Committee (IACUC).

Both male and female mice were used in each experiment unless stated otherwise. Colonies were maintained and genotyped according to The Jackson Laboratory guidelines. Mice were group-housed, and their health was monitored daily for signs of illness (excluding age-related weight loss). Mice were euthanized immediately upon reaching the clinical endpoint, as determined by the staff of veterinary and biological services.

### In vivo treatments

Dasatinib (LC Laboratories, #D-3307; 5 mg/kg) and quercetin (USP, #1592409; 50 mg/kg) were administered orally (gavage). Control mice were administered an equal volume of vehicle (30% polyethylene glycol, 60% phosal and 10% ethanol). Ganciclovir (GCV) (Sigma-Aldrich, #G2536-100MG; 25 mg/kg) was injected intraperitoneally (i.p.). DBA/2-mdx and DBA/2-mdx^p16−3MR^ mice were administered dasatinib plus quercetin (D + Q) or GCV, respectively, twice a week for 2–4 months, as indicated in the figure legends. The specific treatment timelines were selected based on the quantitative burden of senescent cells rather than on chronological age.

### Muscle force measurement

Ex vivo force measurements of extensor digitorum longus (EDL) muscles were assessed as previously described [5, 17]. Briefly, mice were euthanized, and muscles were immediately excised and placed into a dish containing oxygenated Krebs-Henseleit solution. Muscles were mounted vertically in a temperature-controlled chamber and immersed in a continuously oxygenated Krebs-Ringer bicarbonate buffer solution, with 10 mM glucose. One end of the muscle was linked to a fixed clamp, while the other end was connected to the lever arm of a force transducer (300B, Aurora Scientific) using a nylon thread. The optimum muscle length was determined from micromanipulations of muscle length to produce the maximum isometric twitch force. The maximum specific isometric tetanic force was determined from the plateau of the curve of the relationship between specific isometric force, with a stimulation frequency ranging from 1 to 250 Hz. Force was normalized per muscle area, determined by dividing the muscle mass by the product of length and muscle density of 1.06 mg/mm^3^, to calculate the specific force (mN/mm^2^).

### Grip strength

Grip strength was measured with a computerized grip strength meter. The apparatus consisted of a T-shaped metal bar connected to a force transducer. Mice were allowed to grasp the metal bar of the force transducer with their forepaws. Once a firm grip was established, the experimenter gently pulled the animal backwards by the tail until the grip was released. The maximum force of each measurement was automatically recorded in grams (g) by the device. The three highest values were averaged.

### Cardiac function

Transthoracic echocardiography was performed under light sedation (1% isoflurane in O_2_) prior to euthanasia. A digital ultrasound system (VisualSonics Vevo2100 High-Resolution In Vivo Imaging System; FUJIFILM VisualSonics, Toronto, Canada) equipped with a 55 MHz linear-array transducer was used for image acquisition. Standard parasternal long-axis and short-axis views were obtained in B-mode and M-mode, and functional parameters were measured. All images were acquired and analyzed by an investigator blinded to the treatment groups. Conventional systolic functional parameters were measured, including left ventricular (LV) ejection fraction, LV fractional shortening, stroke volume, cardiac output, LV anterior and posterior wall thickness, LV inner diameter, LV mass, sphericity index (SI), and the relative wall thickness (RWT). Diastolic function was assessed using the apical four-chamber view by a pulsed-wave Doppler at the level of the mitral valve inflow. Early (E) and late (A) diastolic filling velocities (corresponding to passive and atrial-driven ventricular filling, respectively) were measured, and the E/A ratio was calculated. Mitral deceleration time and mitral in-flow velocity time integral (VTI) were analyzed to further evaluate LV diastolic function.

### p16-3MR Renilla luciferase reporter assay

Renilla luciferase activity was measured in tibialis anterior (TA) muscles from p16-3MR and DBA/2-mdx^p16−3MR^ mice. Anesthetized mice were injected intramuscularly with coelenterazine H (PerkinElmer, #760506) and immediately subjected to measurement with the IVIS Lumina III (PerkinElmer).

### Human biopsies

DMD human samples were provided by Dr. J. Colomer (Hospital Sant Joan de Déu, Barcelona, Spain); DMD diagnosis was established based on the total absence of dystrophin by immunohistochemistry and confirmed by Western blotting. Muscle samples were obtained by a standard quadriceps muscle biopsy from eight individuals with DMD (aged 6 to 12 years) and six healthy control individuals (aged 9 to 15 years). Histological analysis and characterization of SA-β-gal+ cells were conducted in accordance with the protocols used for mouse specimens, as detailed in the following section.

### Muscle and heart histology

TA, diaphragm and heart ventricles were embedded in OCT solution (TissueTek, #4583), frozen in isopentane cooled with liquid nitrogen, and stored at -80 °C until analysis. Heart, diaphragm and TA cryosections (10-µm thick) were collected and stained for SA-β-gal (AppliChem, #A1007,0001), hematoxylin/eosin (H&E; Sigma-Aldrich, #HHS80 and #45235) or Sirius Red (Sigma-Aldrich, #365548). Quantification of the cross-sectional areas (CSA) of TA and diaphragm fibers in H&E–stained sections, the percentage of muscle or heart area positive for Sirius Red staining, and the number of SA-β-gal+ cells was performed with ImageJ software. Immunofluorescence was performed by the sequential addition of each primary and secondary antibody using positive and negative controls. The sections were air-dried, fixed, washed with PBS and incubated with primary antibodies according to the standard protocol after blocking with a high-protein-containing solution in PBS for 1 h at room temperature. Subsequently, the slides were washed with PBS and incubated with the appropriate secondary antibodies and labelling dyes. Telomere immuno-FISH was performed after γH2AX immunofluorescence with a telomeric PNA probe (Panagene, F1002-5), as described previously [5].

### RNA isolation and RT-qPCR

Total RNA was isolated from snap-frozen TA muscles and heart tissue using miRNeasy Mini Kit (Qiagen, #1038703). DNase digestion was performed using 2U DNase (Qiagen, #1010395). For RT-qPCR, cDNA was synthesized from total RNA using SuperScript VILO (Invitrogen, #11754050) according to the manufacturer’s instructions. qPCR reactions were performed as described previously [5]. Reactions were run in triplicate, and automatically detected threshold cycle values were compared between samples. Transcripts of the *Rpl7* housekeeping gene were used as the endogenous control, with each unknown sample normalized to the *Rpl7* control (see Supplementary Table 1 for the list of primers used).

### Bulk RNA-sequencing

Sequencing libraries were generated from total RNA from TA and heart using the Watchmaker mRNA Library Prep Kit v1.1.0823, and these were sequenced on an Illumina NextSeq 2000 platform (paired-end, 51 bp read length, ~ 250 million reads per run). Bulk RNA-sequencing (RNA-seq) data from TA and heart were processed using the Nextflow nf-core pipeline [18, 19]. Raw reads were trimmed using *Trimgalore* (v.0.6.10) and quantified using *Salmon* (v.1.10.3) and the reference genome GRCm39 [20, 21]. Counts were summarized using *tximport* (v1.30.0), and lowly expressed genes were removed (genes with greater than 5 counts in 3 or more samples were retained) [22]. Surrogate variable analysis (SVA) was applied to the TA data to remove hidden batch effects, with one estimated SV added to the DESeq2 design formula [23, 24]. For heart data, sex was added to the DESeq2 formula as a known batch. Differentially expressed genes were computed using DESeq2, and genes were considered significant if they had adjusted *p* < 0.05 and absolute log2 fold change > 0.5. PCA plots correcting for known and hidden batches were produced by running PCA on corrected counts from limma’s removeBatchEffect [25].

GO pathway enrichment of DEGs was performed using the *hyperGTest* from the package *GOstats *[26] (v2.68.0) with “conditional = TRUE” and “ontology = BP”. Over-representation analyses of DEGs in select muscle gene sets from SysMyo were performed using the *phyper* function. For both enrichment analyses, the Benjamini-Hochberg p-value adjustment using the function *p.adjust* was applied.

### Genotyping of mice

The following primer sequences were used for PCR-based genotyping: p16-3MR-1 (5′-AACGCAAACGCATGATCACTG-3′) and p16-3MR-2 (5′-TCAGGGATGATGCATCTAGC-3′). Mice carrying the p16-3MR transgene were identified by the presence of a distinct amplification product of 202 base pairs (bp).

### Statistical analysis

GraphPad Prism software was used for all statistical analyses except for sequencing data analysis. Quantitative data displayed as histograms are expressed as mean ± standard error of the mean (represented as error bars). Results from each group were averaged and used to calculate descriptive statistics. Mann–Whitney test (two-tailed) was used for comparisons between groups otherwise specified in the figure legends. For comparisons in the force-frequency curves, 2-way ANOVA was applied. For group comparisons of echocardiographic parameters, unpaired Student’s *t*-test was used. Statistical significance was set at *p* < 0.05.

## Results

### Senescent cells accumulate in the skeletal muscle of dystrophic mice at early disease stages

**Fig. 1:**
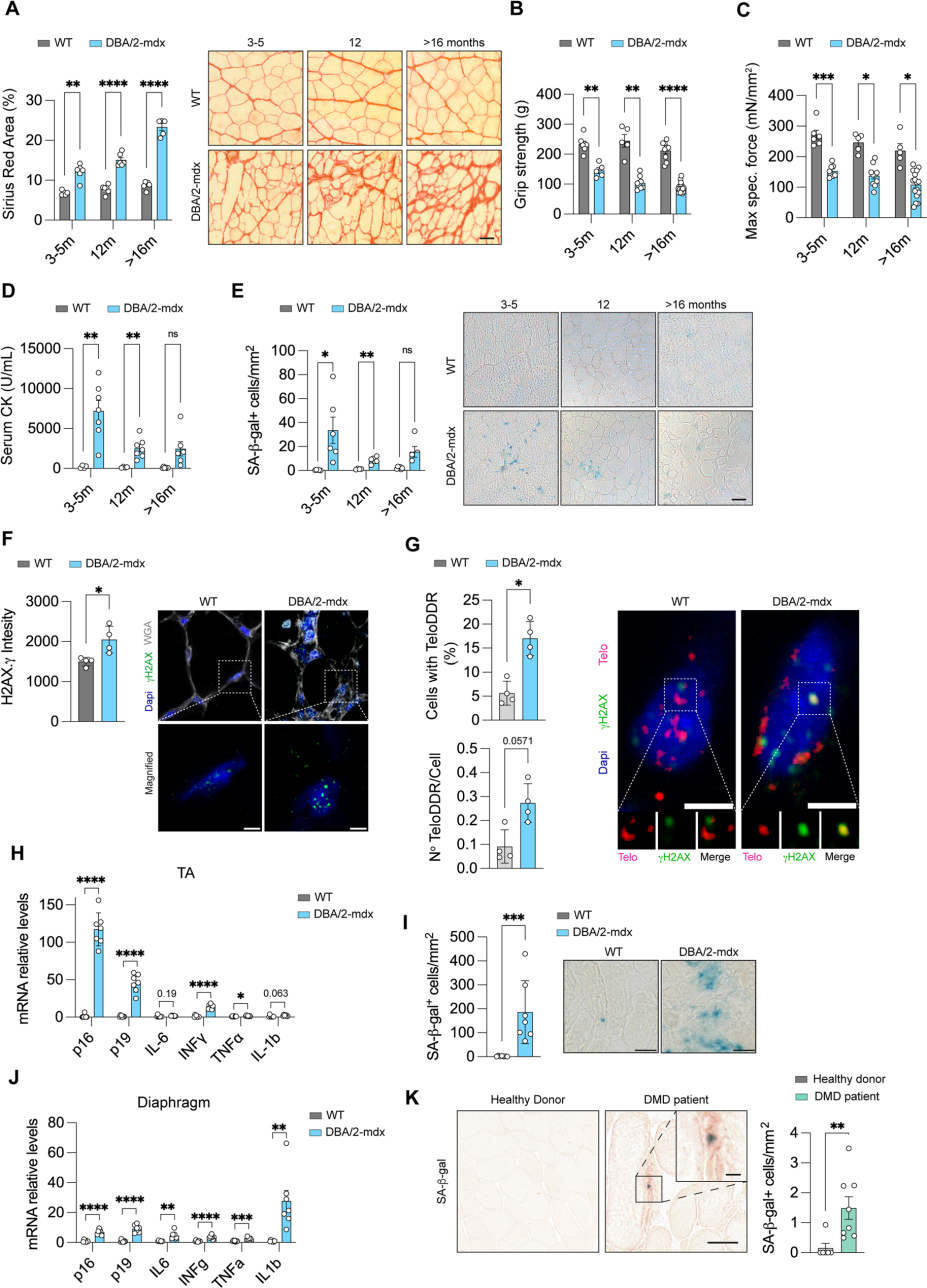
Senescent cells accumulate in the dystrophic skeletal muscle of young DBA/2-mdx mice and DMD patients. **A** Quantification of collagen deposition in the tibialis anterior (TA) muscle of wild-type (WT) and DBA/2-mdx mice at the indicated ages (left), and representative images of Sirius Red staining (right). **B **Four-limb grip strength of wild-type (WT) and DBA/2-mdx mice. **C** Maximum specific force of the extensor digitorum longus (EDL) muscle. **D** Serum creatine kinase (CK) levels in WT and DBA/2-mdx mice across age groups. **E** Quantification and representative images of SA-β-gal⁺ cells in the TA muscle of WT and DBA/2-mdx mice across age groups. **F** Quantification and representative images of DNA damage by γH2AX intensity in extra-myofiber nuclei of TA from WT and DBA/2-mdx mice at young ages. **G** Quantification and representative images of percentage of cells with telomeric DNA damage response (TeloDDR, upper graph) and average number of TeloDDR foci (bottom graph) in extra-myofiber nuclei of TA from WT and DBA/2-mdx mice at young ages. **H** Relative RNA levels of senescent markers in TA of WT and DBA/2-mdx mice at young ages assessed by RT-qPCR. **I** Quantification and representative images of SA-β-gal⁺ cells in the Diaphragm muscle of WT and DBA/2-mdx mice at 3–5 months of age (young). **J** Relative RNA levels of senescent markers in the diaphragm of WT and DBA/2-mdx mice at young ages assessed by RT-qPCR. **K** SA-β-gal⁺ cells in muscle biopsies from individuals with DMD. Data are presented as mean ± SEM. The Mann–Whitney test was used for all comparisons (in **A**-**E**, individual comparisons were performed between age-matched WT and DBA/2-mdx mice). Statistical significance is indicated by *p < 0.05; **p < 0.01; ***p < 0.001; and ****p < 0.0001. Scale bars, 50 μm in (**A**,** E**); 2.5 μm in (**F**,** G**); 50 μm in (**K**) (10 μm in amplified image)

The DBA/2-mdx mouse is a well-characterized animal model for studying DMD. Compared to other mdx strains (e.g., C57BL/10-mdx), it more accurately recapitulates several key features of human disease [27], including reduced hindlimb muscle mass, fewer myofibers, and increased fibrosis and fat accumulation [28, 29]. In this strain, fibrosis in skeletal muscle progressively increases over time, reaching a peak after 16 months of age (Fig. 1A). Muscle strength measured by grip test (Fig. 1B) and by ex vivo force generation of the EDL muscle (Fig. 1C) decreases with age and remains lower in DBA/2-mdx mice than in DBA/2 wild-type mice (WT) at all stages. Serum creatine kinase (CK) levels, an indicator of muscle damage, were elevated during the early stages of the disease (3–5 months of age) but declined later during disease progression. This suggests active muscle degeneration early on with a later shift to fibrosis, consistent with previous reports [28, 29] (Fig. 1D). Correspondingly, SA-β-gal⁺ cells were more abundant in the TA muscles of DBA/2-mdx mice at early disease stages. Although still present, their number declined with age (Fig. 1E), paralleling CK levels and indicating reduced degeneration–regeneration cycles and increased fibrosis. Moreover, muscles of DBA/2-mdx mice exhibited higher total DNA damage response (DDR) and elevated telomeric DDR (TeloDDR) than WT mice at young ages (Fig. 1F–G). DBA/2-mdx mice also showed increased mRNA levels of various senescence markers, including the cyclin-dependent kinase inhibitors p16 and p19, as well as pro-inflammatory factors associated with the SASP, such as IL-6, IFNγ, TNFα, and IL-1β (Fig. 1H).

DBA/2-mdx mice also reproduce the human DMD fibrotic phenotype in the diaphragm muscle over time [30, 31], which ultimately leads to impaired respiratory function. The diaphragm muscle of DBA/2-mdx mice also exhibited a high number of SA-β-gal⁺ cells and elevated levels of senescence markers at young ages (Fig. 1I, J). In contrast, the number of SA-β-gal⁺ cells in soleus muscle was lower than in the TA and diaphragm muscle (Supplementary Fig. 1A). Similarly, other commonly used senescence markers were reduced in soleus muscle of DBA/2-mdx mice compared to WT at young ages (Supplementary Fig. 1B). These data suggest that senescent cell accumulation in slow-twitch muscles follows a different pattern than in muscles rich in fast-twitch fibers (TA and diaphragm) in the DBA/2-mdx model.

Notably, skeletal muscle biopsies from individuals with DMD also showed increased numbers of SA-β-gal⁺ cells compared with healthy controls, indicating that senescent cells are a feature of both the mdx mouse model and human DMD (Fig. 1K).

### Senescent cell clearance improves dystrophic muscle phenotype in young mice

To investigate the impact of senescent cells on the progression of muscular dystrophy at different disease stages, we treated young (3 months), adult (12 months) and old (> 16 months) DBA/2-mdx mice twice weekly for 8 weeks with either the senolytic cocktail D + Q or vehicle (as a control).

**Fig. 2:**
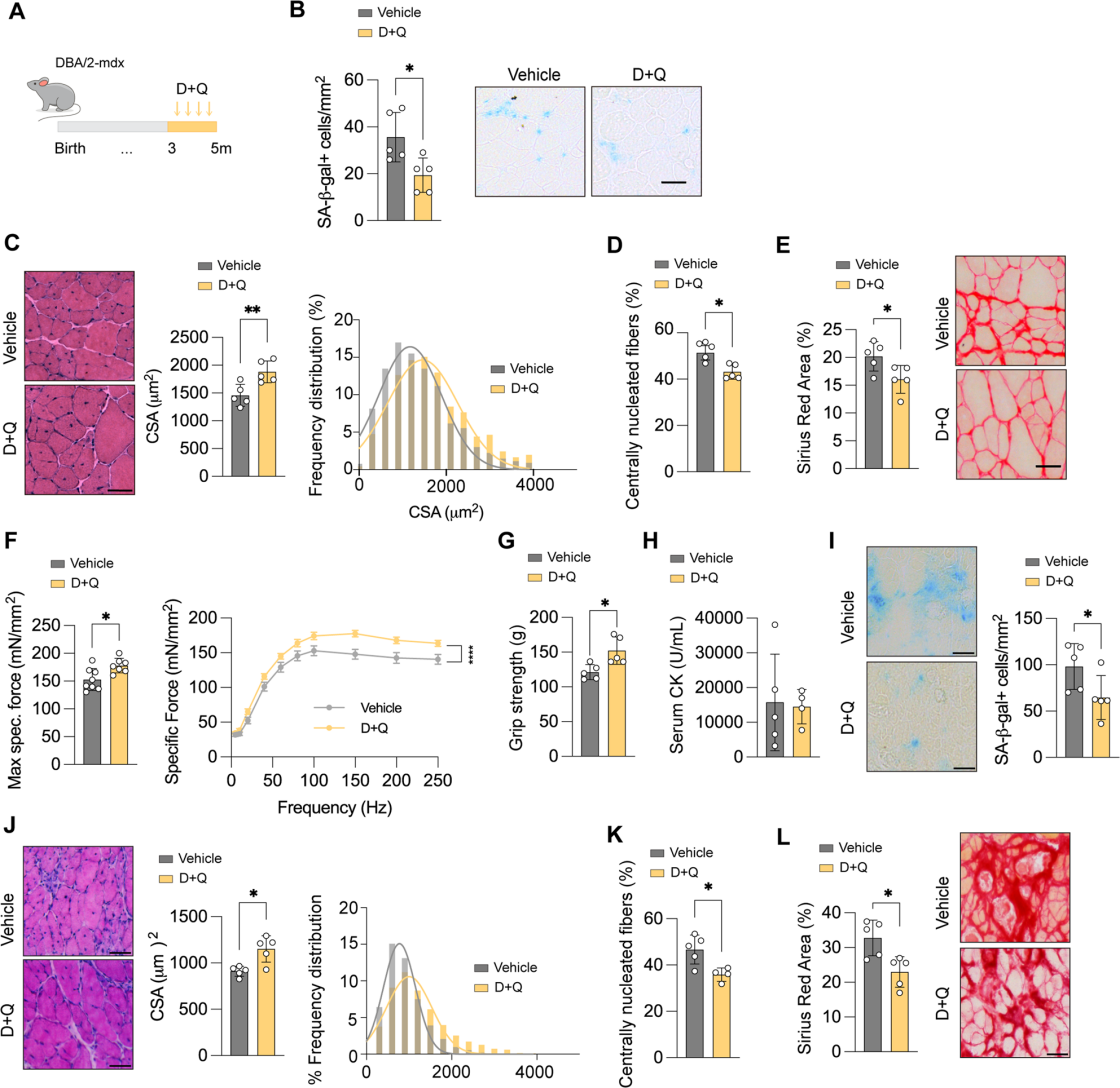
Pharmacological reduction of senescent cells improves skeletal muscle function in young dystrophic mice. **A** Three-month-old DBA/2-mdx mice were treated with vehicle or D + Q twice weekly for 8 weeks. **B–E** Histology from TA; Histology from diaphragm is from **I**-**L**. **B** Quantification and representative images of SA-β-gal⁺ cells in the TA muscle of DBA/2-mdx mice. **C** Mean cross-sectional area (CSA) of fibers and frequency distribution of muscle fiber size in the TA of DBA/2-mdx mice based on H&E-stained sections. **D** Percentage of centrally nucleated fibers in the TA based on H&E-stained sections. **E** Quantification of collagen deposition and representative Sirius Red staining images. **F** Maximum specific force and force–frequency curves of the EDL muscle in vehicle and D + Q–treated mice. **G** Four-limb grip strength. **H** Serum creatine kinase (CK) levels in DBA/2-mdx mice treated with vehicle or D + Q. **I** Quantification and representative images of SA-β-gal⁺ cells in the diaphragm of DBA/2-mdx mice. **J** Mean cross-sectional area (CSA) of fibers and frequency distribution of muscle fiber size in the diaphragm of DBA/2-mdx mice based on H&E-stained sections. **K** Percentage of centrally nucleated fibers in the diaphragm based on H&E-stained sections. **L** Quantification of collagen deposition and representative Sirius Red staining images. Data are presented as mean ± SEM. The Mann–Whitney test was used for all comparisons. Two-way ANOVA was used for the force-frequency curve analysis. Statistical significance is indicated by *p < 0.05; **p < 0.01; ***p < 0.001; and ****p < 0.0001. Scale bars, 50 μm

In young mice, D + Q treatment for 2 months reduced the number of SA-β-gal^+^ cells in the TA muscle compared to vehicle-treated controls (Fig. 2A-B). This reduction was accompanied by increased myofiber cross-sectional areas (CSA) (Fig. 2C), a reduced percentage of centrally nucleated fibers (Fig. 2D), decreased fibrosis (Fig. 2E) and functional gains in specific force of the EDL muscle and grip strength (Fig. 2F-G). Similar to its effects in limb muscles, D + Q treatment reduced SA-β-gal⁺ cells in the diaphragm (Fig. 2I), showing increased CSA (Fig. 2J), a lower percentage of centrally nucleated fibers (Fig. 2K) and reduced fibrosis (Fig. 2L). Despite the histological and functional improvements reported in skeletal muscle after D + Q treatment, CK levels in serum did not change compared to vehicle-treated mice (Fig. 2H). Of note, in adult and old mice, the therapeutic effects of senescent cell clearance were progressively less pronounced, correlating with their lower abundance (Supplementary Fig. 2). Reduced fibrosis was only present in the adult mice but not at later disease stages, although a trend towards a functional benefit was also observed in the old D + Q-treated mice (Supplementary Fig. 2).

D + Q treatment reduced the percentage of proliferating satellite cells (Pax7⁺/Ki67⁺) without affecting Pax7⁺/MyoD⁺ cells, indicating that early satellite cell activation and myogenic commitment are preserved (Supplementary Fig. 3). The accompanying reduction in centrally nucleated fibers suggests that newly formed fibers mature more rapidly. Together, these results indicate that senolytic treatment enhances regenerative efficiency by accelerating fiber maturation rather than increasing the initiation of myogenesis.

**Fig. 3:**
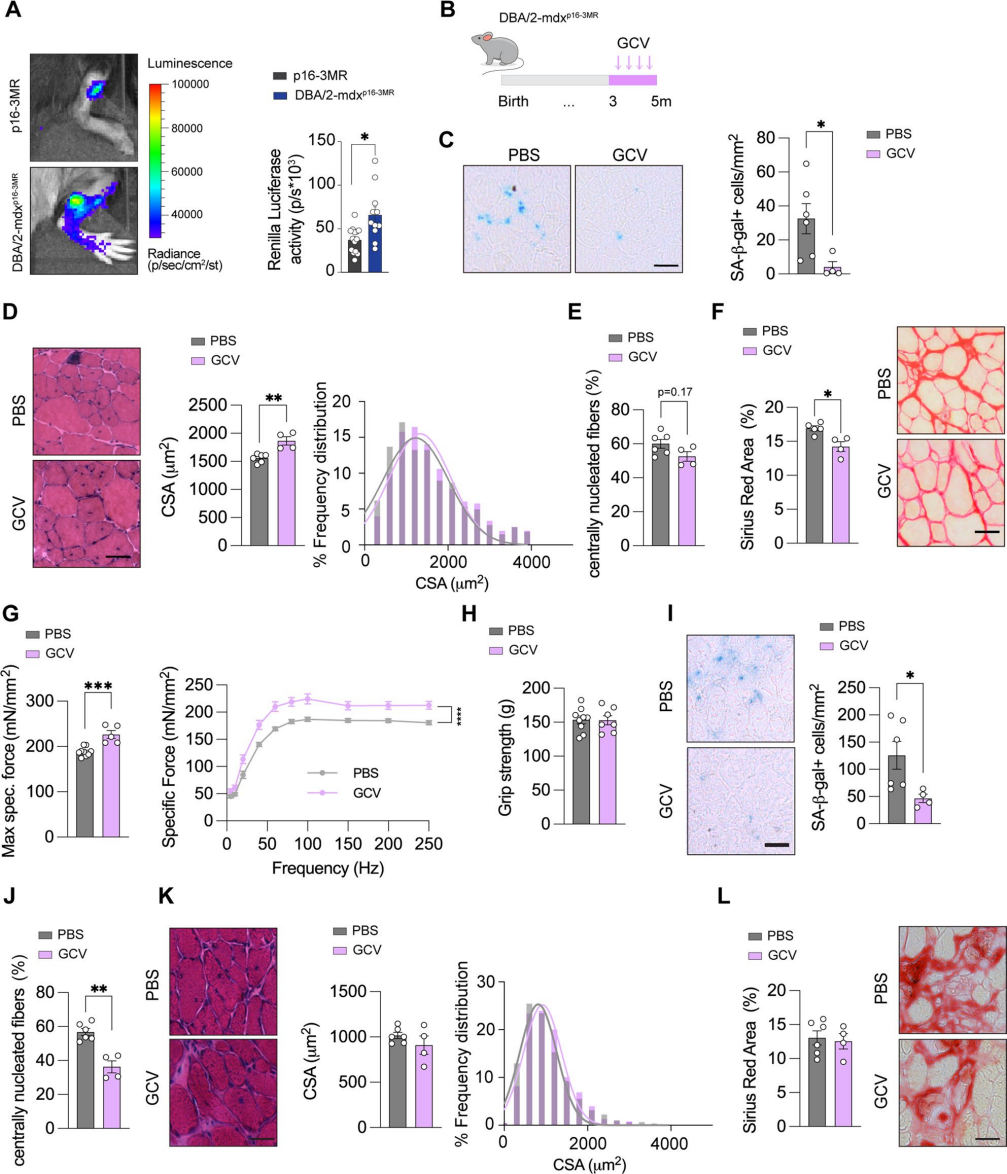
Genetic ablation of senescent cells improves skeletal muscle function in DBA/2-mdx^p16−3MR^ dystrophic mice. **A** Luciferase activity in the TA muscles of young (3–5 months) p16-3MR and DBA/2-mdx^p16−3MR^ mice. **B** Three-month-old DBA/2-mdx^p16−3MR^ mice were treated with PBS or GCV twice weekly for 8 weeks. **C–F** Histology from TA; (**I–L**), histology from diaphragm. **C** Quantification and representative images of SA-β-gal⁺ cells in the TA. **D** Mean cross-sectional area (CSA) of fibers and frequency distribution of muscle fiber size based on H&E-stained sections. **E** Percentage of centrally nucleated fibers in the TA based on H&E-stained sections. **F** Quantification of collagen deposition and representative Sirius Red staining images in the TA. **G** Maximum specific force and force–frequency curves of the EDL muscle in PBS and GCV–treated mice. **H** Four-limb grip strength. **I** Quantification and representative images of SA-β-gal⁺ cells in the diaphragm of DBA/2-mdx^p16−3MR^ mice. **J** Percentage of centrally nucleated fibers in the diaphragm based on H&E-stained sections. **K** Mean cross-sectional area (CSA) of fibers and frequency distribution of muscle fiber size in the diaphragm of DBA/2-mdx^p16−3MR^ mice based on H&E-stained sections. **L** Quantification of collagen deposition and representative Sirius Red staining images. Data are presented as mean ± SEM. The Mann–Whitney test was used for all comparisons. Two-way ANOVA was used for the force-frequency curve analysis. Statistical significance is indicated by *p < 0.05; **p < 0.01; ***p < 0.001; and ****p < 0.0001. Scale bars, 50 μm

To further investigate the presence and role of senescent cells in dystrophic muscles, we crossed the DBA/2-mdx strain with the p16-3MR mouse [16], which enables visualization of p16^INK4a^ expressing cells through the expression of a synthetic Renilla luciferase reporter and allows the selective elimination of these cells following GCV treatment in vivo. The resulting DBA/2-mdx^p16−3MR^ mice showed increased luciferase activity in the TA muscle compared to control DBA/2^p16−3MR^ mice (Fig. 3A). DBA/2-mdx^p16−3MR^ mice were treated with GCV during the early disease phase (3–5 months), following the same regimen as D + Q (twice weekly for 8 weeks) (Fig. 3B). Similar to the senolytic treatment, GCV decreased senescent cell numbers (Fig. 3C), resulting in increased CSA (Fig. 3D), a slightly lower percentage of centrally nucleated fibers (Fig. 3E), reduced fibrosis (Fig. 3F) and improved muscle specific force of the EDL muscle and grip strength (Fig. 3G–H). Although the number of SA-β-gal⁺ cells was reduced in the diaphragm of these mice (Fig. 3I) and the percentage of centrally nucleated fibers decreased (Fig. 3J) after GCV treatment, no significant differences in fiber size or fibrosis deposition were detected (Fig. 3K and L). As in the DBA/2-mdx^p16−3MR^ mice GCV only targets the p16 ^INK4a^ cells, the different outcomes seen TA and diaphragm after GCV treatment could be due to the differences in p16 expression in both muscles, which was higher in the TA (Supplementary Fig. 4).

**Fig. 4:**
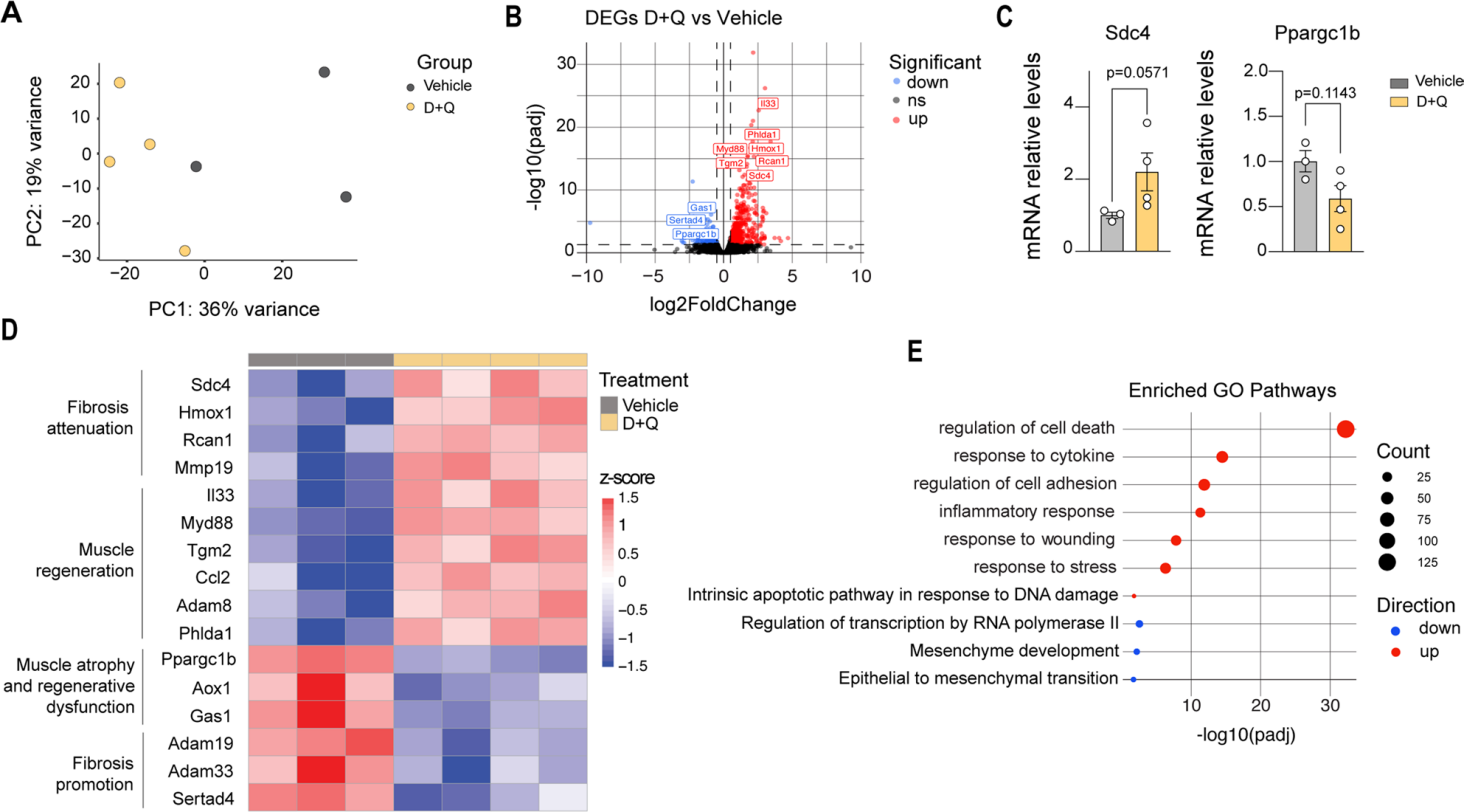
Transcriptomic changes in TAs of DBA/2-mdx mice after D + Q treatment for 2 months in young mice. **A** Principal component analysis (PCA) plot of gene expression data of TAs from vehicle- and D + Q–treated mice. **B** Volcano plot of differentially expressed genes (DEGs; adj *p* < 0.05 and log2FoldChange > 0.5) comparing D + Q– vs. vehicle-treated TAs. **C** qPCR levels of selected genes upregulated or downregulated in the RNA-seq. **D** Heatmap showing selected up- and downregulated DEGs in the D + Q group related to muscle-relevant functions. **E** Dot plot representing selected enriched GO pathways in D + Q vs. vehicle TAs. In (C), data are presented as mean ± SEM. The Mann–Whitney test was used for all comparisons. Statistical significance is indicated by *p < 0.05

To investigate the molecular effects of senescent cell reduction in young dystrophic mice, we conducted bulk RNA-seq on TA muscles. D + Q– and vehicle-treated groups were transcriptionally distinct, separating along the first principal component in the principal component analysis (PCA) plot (Fig. 4A). A total of 549 and 212 differentially expressed genes (DEGs) were up- and downregulated, respectively, in D + Q–treated muscle compared with controls (Fig. 4B). Levels of selected up- and downregulated genes were assessed by qPCR to corroborate the sequencing data (Fig. 4C). Among the most significant upregulated DEGs were genes associated with muscle function and regeneration (*Il33*,* Myd88*,* Tgm2*,* Ccl2*,* Adam8* and *Phlda1*) [32–37] and fibrosis attenuation (*Sdc4*,* Hmox1*,* Rcan1*,* Mmp19*) [38–41] (Fig. 4D). The most significant downregulated DEGs included genes associated with muscle atrophy (*Ppargc1b*,* Aox1*) [42, 43], regenerative dysfunction (*Gas1*) [44] and fibrosis (*Adam19*,* Adam33*,* Sertad4*) [45–47] (Fig. 4D). Collectively, these results indicate that senescent cell reduction in dystrophic mice promotes muscle health through multiple pathways, including the decreased expression of deleterious and pro-fibrotic genes and increased expression of genes involved in muscle repair and fibrosis attenuation. These molecular changes correlate with the histological findings (Fig. 2) that showed that muscle from D + Q–treated mice exhibited reduced fibrosis deposition and a lower percentage of centrally nucleated regenerating fibers. Notably, other enriched pathways seen in our RNA-seq data are similar to those reported by Novais and colleagues [48] after D + Q treatment in the intervertebral disc degeneration model, including upregulation of cell death regulation, cell adhesion and response to stress, and downregulation of processes related to RNA transcription (Fig. 4E).

### Reduction of senescent cells in adult dystrophic mice improves cardiac pathology

**Fig. 5:**
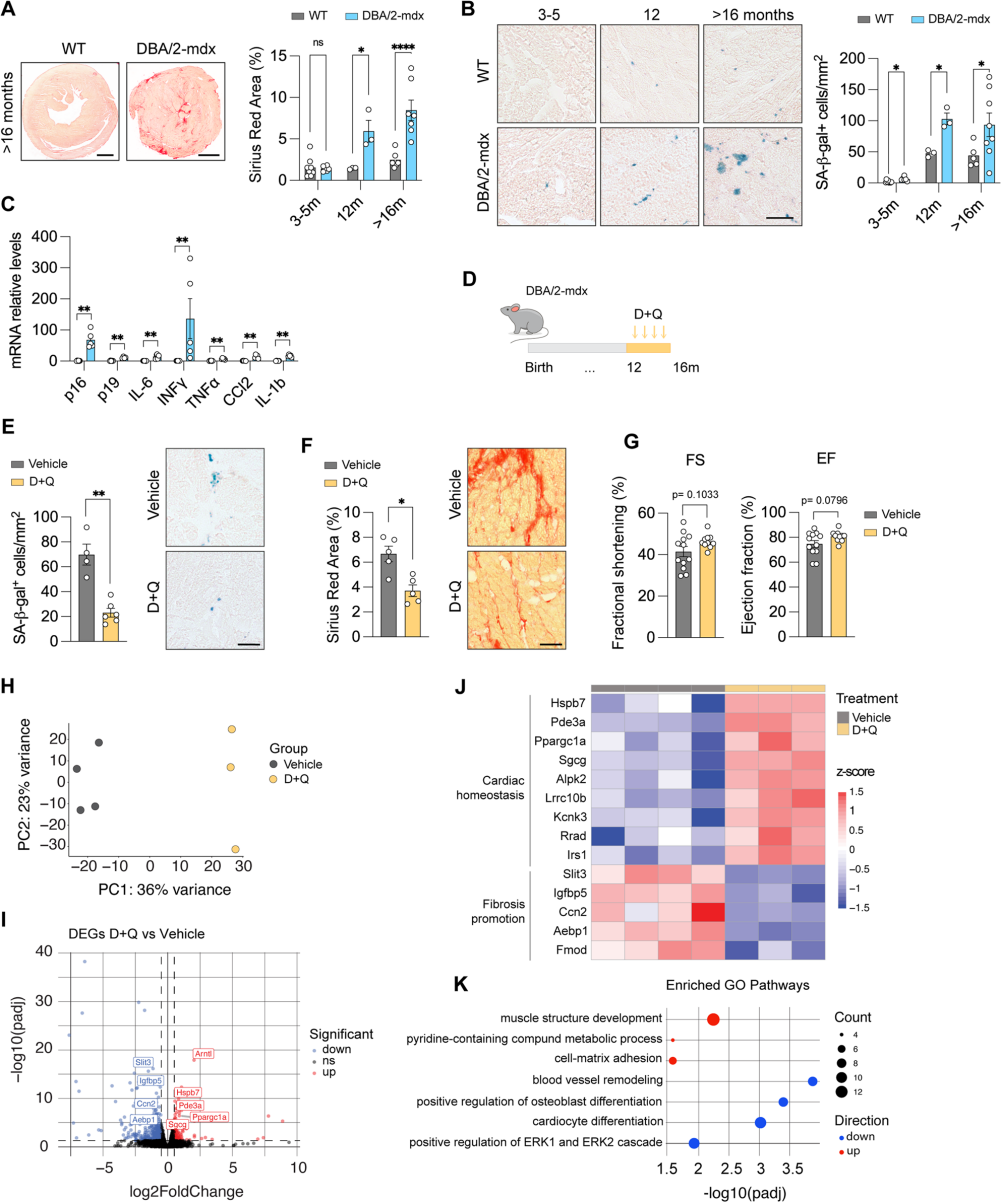
Senolytic treatment improves heart pathology in dystrophic mice at advanced disease stages. **A** Collagen quantification by Sirius Red staining in the hearts of wild-type (WT) and DBA/2-mdx mice at the indicated ages. Representative images of aged hearts from both genotypes are shown. **B** SA-β-gal⁺ cell number and representative pictures of heart sections from wild-type (WT) or DBA/2-mdx mice at each age group. **C** Relative RNA levels of senescent markers in the hearts of WT and DBA/2-mdx mice at adult ages (12 months) assessed by RT-qPCR. **D** 12-month-old DBA/2-mdx mice were treated twice weekly for 4 months with D + Q or vehicle. **E** Quantification of SA-β-gal⁺ cells and representative images of the hearts after treatment with vehicle or D + Q. **F** Collagen deposition in hearts from mice treated with vehicle or D + Q. **G** Fractional shortening (FS) and ejection fraction (EF) of hearts assessed by echocardiography. Data are presented as mean ± SEM. The Mann–Whitney test was used for all comparisons (in **A** and **B**, individual comparisons were performed between age-matched WT and DBA/2-mdx mice). The unpaired Student’s *t*-test was used in (**F)**. Statistical significance is indicated by *p < 0.05; **p < 0.01; ***p < 0.001; and ****p < 0.0001. Scale bar in (**A**) is 1 mm. Scale bars in (**B**, **E**, **F**), 50 μm. **H** Principal component analysis (PCA) plot of gene expression data of hearts from mice treated with vehicle or D + Q. **I** Volcano plot of differentially expressed genes (DEGs; adj *p* < 0.05 and |log2FoldChange| > 0.5) comparing D + Q– and vehicle-treated hearts. **J** Heatmap showing selected up- or downregulated DEGs in the D + Q group related to cardiac-relevant functions. **K** Dot plot representing selected enriched GO pathways in D + Q vs. vehicle hearts

The DBA/2-mdx hearts showed a progressive increase in fibrosis (Fig. 5A) and accumulation of SA-β-gal⁺ cells with age (Fig. 5B). Unlike skeletal muscle, cardiac muscle did not exhibit senescent cells at young ages (3 to 5 months) (Fig. 5B). The hearts of WT mice exhibited an age-related increase in SA-β-gal⁺ cells, although the number of these cells was consistently higher in DBA/2-mdx hearts (Fig. 5B). Notably, a higher abundance of SA-β-gal⁺ cells was observed in the ventricles compared with the atria (data not shown), suggesting regional variation possibly due to increased mechanical and physiological stress. Elevated mRNA levels of various senescence markers, including the cyclin-dependent kinase inhibitors p16 and p19, as well as pro-inflammatory factors associated with the SASP (IL-6, IFNγ, TNFα, and IL-1β), were observed in the hearts of adult DBA/2-mdx mice compared to WT (Fig. 5C).

As the greatest therapeutic benefit of senolysis in skeletal muscle was observed when the senescent cell burden was highest, we treated aged DBA/2-mdx mice (12 months) with D + Q twice weekly for four months to evaluate its effects on cardiac pathology (Fig. 5D). D + Q treatment significantly reduced the number of SA-β-gal⁺ cells (Fig. 5E) and decreased cardiac fibrosis (Fig. 5F). Echocardiography at the end of the treatment period also revealed a trend toward functional improvement, reflected by slight increases in the ejection fraction (EF; the percentage of blood ejected from the ventricle during systole) and fractional shortening (FS; the percentage reduction in ventricular diameter from diastole to systole) in D + Q–treated mice (Fig. 5G, Supplementary Table 2). The modest improvements in EF and FS, which reflect ventricular function, may be explained by the higher burden of senescent cells in the ventricles compared with the atria in the DBA/2-mdx model and may reflect the need of longer treatments to improve heart function in addition of the morphological improvement.

Bulk RNA-seq of cardiac tissue from DBA/2-mdx mice treated with vehicle or D + Q for four months showed distinct transcriptional profiles in the PCA plot (Fig. 5H), with 177 genes upregulated and 386 downregulated in D + Q-treated mice (Fig. 5I). Several of the most significantly upregulated DEGs play key roles in maintaining cardiac function, particularly contractile performance, by regulating cardiomyocyte mechanotransduction, preserving intercalated disc integrity and modulating actin cytoskeleton organization (e.g., *Hspb7*,* Pde3a*,* Ppargc1a*,* Sgcg*,* Alpk2*,* Lrrc10b*,* Kcnk3*,* Rrad*,* Irs1*, and *Ctnnal1*) [49–60] (Fig. 5I).

Analogous to the TA results, the top downregulated DEGs included pro-fibrotic genes (*Slit3*,* Igfbp5*,* Ccn2*,* Aebp1*,* Fmod60) *[61–65] (Fig. 5J). Other enriched pathways in hearts of D + Q-treated mice included upregulation of muscle structure development, metabolic process and cell-matrix adhesion (Fig. 5K). Collectively, these results demonstrate that senescent cell clearance reduced cardiac fibrosis and activated transcriptional programs that promote cardiac function and reduce fibrosis, providing molecular support for the beneficial effects of D + Q in dystrophic hearts.

### Senolysis modulates similar pathways in cardiac and skeletal muscle of dystrophic mice

**Fig. 6:**
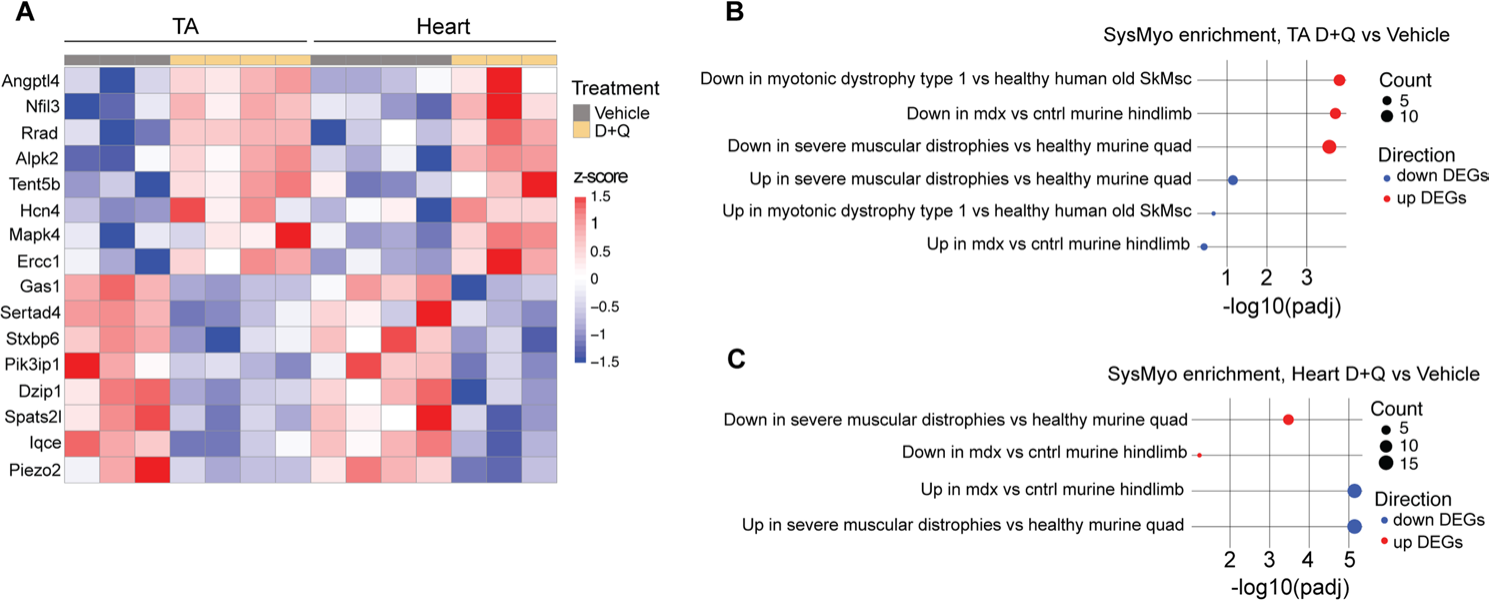
D + Q treatment in dystrophic mice activates a similar transcriptomic signature in skeletal muscle and heart. **A** Heatmaps showing the expression of shared up- and downregulated DEGs in TA and heart from vehicle- or D + Q–treated DBA/2-mdx mice. Vehicle, gray; D + Q, yellow. **B**, **C** Dot plots showing selected enriched pathways from the SysMyo gene sets in the TA (**B**) and heart (**C**) after D + Q treatment of DBA/2-mdx mice

Comparing the transcriptional signatures of D + Q in the heart and skeletal muscle of dystrophic mice revealed a shared signature containing known modulators of skeletal and cardiac muscle health. Shared upregulated DEGs between the heart and skeletal muscle included genes associated with cardiac homeostasis (e.g., *Alpk2*,* Hcn4*) [55, 66] and DNA repair (*Ercc1*) [67] (Fig. 6A). Downregulated DEGs included genes associated with defective muscle regeneration via MuSC dysfunction (*Gas1*) [68] and inhibition of MuSC activation (*Pik3ip1*) [69] (Fig. 6A). *Piezo2* and *Sertad4* were also downregulated in both tissues after D + Q treatment; both genes are involved in fibroblast activation and their differentiation into myofibroblasts [70, 71]. Overall, even with distinct muscle tissues and treatment timings, D + Q treatment induced a common transcriptional signature in both the heart and TA, reflecting the beneficial effects of senescent cell reduction in dystrophic mice.

Finally, over-representation analysis with the SysMyo gene sets, which include signatures of muscle pathology and physiology [72], showed that DEGs in the D + Q group for both TA and the heart were enriched for genes typically downregulated in mdx or other muscular dystrophies (Fig. 6B, C; Supplementary Fig. 5). This finding aligns with the observed histological and functional improvements seen in TA (Fig. 2B–G) and the heart (Fig. 5).

## Discussion

Our results demonstrate that senescent cells accumulate in both skeletal and cardiac muscles of DBA/2-mdx mice and exert detrimental effects in both tissues, as evidenced by the histological and functional improvements observed following their clearance. Senescent cell accumulation in dystrophic striated muscles follows a distinct trajectory throughout life, appearing early in skeletal muscle and later in cardiac muscle. These findings underscore the importance of temporally optimized therapeutic strategies targeting cellular senescence in muscular dystrophy.

Consistent with this concept, the therapeutic benefits of senescent cell clearance are highly age- and tissue-dependent in mice, with the greatest improvements observed when the senescent cell burden is at its peak. While young skeletal muscle responded robustly to senolytic treatment, the same intervention yielded smaller effects at later stages, in line with previous reports that the regenerative capacity of dystrophic muscle declines over time [73]. In contrast to skeletal muscle, the heart exhibited maximal responsiveness to senolysis during mid- to late stages of disease progression, underscoring the importance of appropriately timed interventions. This also suggests that the cellular and molecular context—such as the presence of an active regenerative response versus established fibrosis—plays a critical role in determining the efficacy of senescence-targeting interventions.

In skeletal muscle, early clearance of senescent cells increased fiber size and improved muscle strength, consistent with a causal role for senescent cells in impairing skeletal muscle regeneration [5, 8, 10, 11]. These data are supported by skeletal muscle transcriptomic profiles, whereby D + Q–treated muscle had reduced expression of pro-fibrotic genes and increased expression of genes regulating muscle repair and fibrosis attenuation compared with control muscle. In contrast to skeletal muscle, senescent cells in the heart peaked around 12 months, and their removal in adult mice reduced fibrosis and downregulated ECM and inflammation pathways, as shown by histology and transcriptomic analysis. Unfortunately, stratification of the available human data from dystrophic patients by age was not feasible due to the limited sample size, the narrow age range and the inherent histological variability among biopsies. Despite these limitations, the human biopsy data and previously published studies [74] provide supportive evidence for the presence of senescent cells in diseased human muscle, complementing our experimental findings in mdx mice.

DMD causes early myocardial injury that advances to dilated cardiomyopathy, whereby fibrosis impairs heart function and increases arrhythmia risk and sudden cardiac death in young individuals [75–77]. Cardiomyocytes in DMD exhibit membrane instability and dysregulated calcium (Ca²⁺) handling, leading to excessive Ca²⁺ influx. This contributes to increased myocardial stiffness, impairing both contraction and relaxation of the heart muscle [78, 79]. Concurrently, the development of myocardial fibrosis induces compensatory hypertrophy in neighboring cardiomyocytes, disrupting electrical conduction and intercellular coupling and thereby increasing the risk of severe arrhythmias [80, 81]. Although D + Q treatment led to a clear morphological reduction in cardiac fibrosis, echocardiographic parameters did not reach statistical significance. Nevertheless, a trend toward improvement following senolytic treatment was clearly detected. Reductions in fibrosis represent a robust structural improvement, which may precede measurable changes in global cardiac function. Echocardiographic outcomes reflect integrated performance and may require greater remodeling, longer follow-up, or larger cohorts to reach statistical significance [82]. These findings highlight a positive impact of senolytic therapy on cardiac tissue, while emphasizing that functional improvements in this experimental setting remain modest and that earlier or prolonged senolytic intervention may be necessary to fully realize cardiac benefits.

Transcriptomic analysis after senolytic treatment further revealed a downregulation of genes associated with cardiac fibrosis and an upregulation of transcriptional programs that promote cardiac function and reduce fibrosis. Further, the overlapping DEGs between heart and skeletal muscle transcriptomics include genes associated with improved tissue function, reinforcing the beneficial effects of the treatment. Altogether, these findings support a beneficial effect of senolytic treatment on cardiac remodeling in dystrophic mice and align with recent studies implicating cellular senescence in cardiac dysfunction [83], revealing a previously underexplored mechanism contributing to cardiac decline in DMD.

Further studies will be required to determine the precise identity of the senescent cell types in each tissue and the mechanisms by which they contribute to DMD pathology. For example, the predominant senescent cell populations in each tissue—whether fibroblasts, endothelial cells, FAPs, or immune cells—remain to be determined. We previously reported that senescent cells exert a detrimental effect on muscle regeneration through their SASP in a model of chemically induced muscle injury [5]. A similar mechanism could operate in dystrophic muscle.

The slightly different effects observed with D + Q and GCV in the same muscles may reflect the distinct senescent cell subpopulations targeted by each approach and their different mechanisms of action. While D + Q targets specific anti-apoptotic pathways, the genetic ablation driven by GCV broadly eliminates cells based solely on high p16^Ink4a^ promoter activity, regardless of the survival mechanism. Consequently, D + Q can clear senescent cells even when they express low levels of p16^Ink4a^ (likely reflecting different senescent cell states), while sparing senescent cells reliant on alternative survival pathways. In contrast, genetic ablation eliminates essentially all p16^Ink4a^ expressing cells, including subpopulations that D + Q may miss, but does not eliminate senescent cells that are p16^Ink4a^ negative and p21^high^. Exploring alternative senolytics or combination therapies may therefore lead to more comprehensive and robust therapeutic outcomes. Notably, D + Q treatment exhibits broad systemic effects that occur independently of senescence clearance. Our current findings are consistent with a senescence-related mechanism, yet off-target actions cannot be completely ruled out. Future studies using complementary genetic or pharmacologic approaches will help clarify these mechanisms.

Finally, longitudinal studies assessing survival, exercise capacity, and long-term cardiac function will be essential to fully evaluate the therapeutic potential of senolytic interventions in DMD. In this context, D + Q is currently being investigated in several clinical trials for aging-related human diseases. Notably, in the context of fibrosis, D + Q treatment has demonstrated functional improvements in patients with idiopathic pulmonary fibrosis in pilot clinical studies [84], providing proof-of-concept evidence that targeting senescent cells (particularly with this senolytic combination) may alleviate functional decline in aging-related diseases. The translation of D + Q therapy to pediatric DMD patients requires careful consideration. Unlike aging-related indications in adults, senescence in DMD is a secondary, disease-driven process, and dosing regimens therefore cannot be directly extrapolated from adult trials. While dasatinib has been safely administered in pediatric oncology patients using body surface area–adjusted dosing [85], the risk–benefit profile, optimal schedule, and long-term safety of intermittent senolytic therapy in children with DMD would require dedicated preclinical studies and early-phase clinical trials. Therefore, our findings should be interpreted as preclinical proof-of-concept rather than immediate therapeutic guidance.

## Conclusions

Our study identifies senescent cells as key contributors to skeletal and cardiac muscle pathology in DMD and demonstrates that their timely clearance can ameliorate disease features in a tissue- and age-dependent manner. These findings position cellular senescence as a modifiable component of the dystrophic niche and support the use of senolytic therapies as a promising adjunct to emerging gene-based treatments for DMD.

## Supplementary Information

Supplementary Material 1: Supplementary Fig. 1. Senescent cell markers in soleus muscles of WT or DBA/2-mdx young mice. (A) Quantification of SA-β-gal⁺ cells and representative images of young WT and DBA/2-mdx mice. (B) Relative mRNA levels of senescent markers in the soleus of WT and DBA/2-mdx mice at young ages assessed by RT-qPCR. Data are presented as mean ± SEM. The unpaired Student's t-test was used in all comparisons (DBA/2-mdx vs WT). Statistical significance is indicated by *p < 0.05; **p < 0.01; ***p < 0.001; and ****p < 0.0001. Scale bar, 50 µm.

Supplementary Material 2: Supplementary Fig. 2. Effects of senolytic treatment in the skeletal muscle of adult and old dystrophic mice. Adult (12 months; A–E) and old (> 16 months; F–J) DBA/2-mdx mice were treated with vehicle or D+Q twice weekly for 8 weeks. (A, F) Quantification and representative images of SA-β-gal⁺ cells in the TA muscle of DBA/2-mdx mice. (B, G) Quantification of collagen deposition and representative images of Sirius Red staining. (C, H) Mean cross-sectional area (CSA) fibers in the TA of DBA/2-mdx mice based on H&E-stained sections. (D, I) Maximum specific force and (E, J) force–frequency curves of the EDL muscle from vehicle- and D+Q–treated mice. The Mann–Whitney test was used for all comparisons. Two-way ANOVA was used for the force-frequency curve analysis. Statistical significance is indicated by *p < 0.05; **p < 0.01; ***p < 0.001; and ****p < 0.0001. Scale bars, 50 µm.

Supplementary Material 3: Supplementary Figure 3. (A) Quantification and representative images of Pax7+/Ki67+ cells in D+Q– and vehicle-treated TAs. (B) Quantification and representative images of Pax7+/MyoD+ cells in D+Q– and vehicle-treated TAs. Yellow arrows show Pax7+/Ki67- nuclei (in A) or Pax7+/MyoD- nuclei (in B); white arrows show Pax7+/Ki67+ nuclei (in A) or Pax7+/MyoD+ nuclei (in B); blue arrows show Pax7-/MyoD+ nuclei. Data are presented as mean ± SEM. The Mann–Whitney test was used for all comparisons. Statistical significance is indicated by *p < 0.05. Scale bars, 50 µm. Scale bars of magnified images, 10 µm.

Supplementary Material 4: Supplementary Fig. 4. p16 mRNA levels in the TA and diaphragm of DBA/2-mdx mice. (A) mRNA levels of p16 in TA and diaphragm of DBA/2-mdx mice relative to age-matched WT mice. (B) mRNA levels of p16 in TA relative to the diaphragm of the same DBA/2-mdx mice. Data are presented as mean ± SEM. An unpaired t-test was used for all comparisons (in A, individual comparisons were performed between age-matched WT and DBA/2-mdx mice). Statistical significance is indicated by ***p < 0.001; and ****p < 0.0001.

Supplementary Material 5: Supplementary Fig. 5. SysMyo DEGs in TA and heart after D+Q treatment of DBA/2-mdx mice. Heatmaps showing the expression of SysMyo genes upregulated and downregulated in the TA (A) and heart (B) after D+Q treatment of DBA/2-mdx mice.

Supplementary Material 6: Supplementary Table 1. DNA Oligos used for RT-qPCR.

Supplementary Material 7: Supplementary Table 2. Cardiac function parameters recorded by echocardiography.

## Data Availability

Further information and requests for resources and reagents should be directed to and will be fulfilled by the lead contact, Dr. Pura Muñoz Cánoves (pmunozcanoves@altoslabs.com). RNA-seq data have been deposited at GEO (accession number GSE324363).

## Abbreviations

CK: Creatine kinase
CO: Cardiac output
CSA: Cross-sectional area
D + Q: Dasatinib plus quercetin
DMD: Duchenne muscular dystrophy
DMSO: Dimethyl sulfoxide
EDL: Extensor digitorum longus
EF: Ejection fraction
E/A: Ratio between E peak and A peak
EDV: End-diastolic volume
ESV: End-systolic volume
FS: Fractional shortening
GCV: Ganciclovir
GSEA: Gene set enrichment analysis
LVAWd: Left ventricular anterior wall in diastole
LVAWs: Left ventricular anterior wall in systole
LVIDd: Left ventricular internal diameter in diastole
LVIDs: Left ventricular internal diameter in systole
LVPWd: Left ventricular posterior wall in diastole
LVPWs: Left ventricular posterior wall in systole
LVm: Left ventricular mass
MuSC: Muscle stem cell
RNA-seq: RNA sequencing
RWT: Relative wall thickness
SASP: Senescence-associated secretory phenotype
SI: Sphericity index
SV: Stroke volume
TA: Tibialis anterior
VTI: Velocity time integral
