# Supplementary figures and images for: Senescence dynamics define therapeutic windows for Duchenne muscular dystrophy in DBA/2-mdx mice
Source: Skelet Muscle. 2026 May 2;16:27. doi: 10.1186/s13395-026-00426-5 (PMC13288804; doi:10.1186/s13395-026-00426-5)

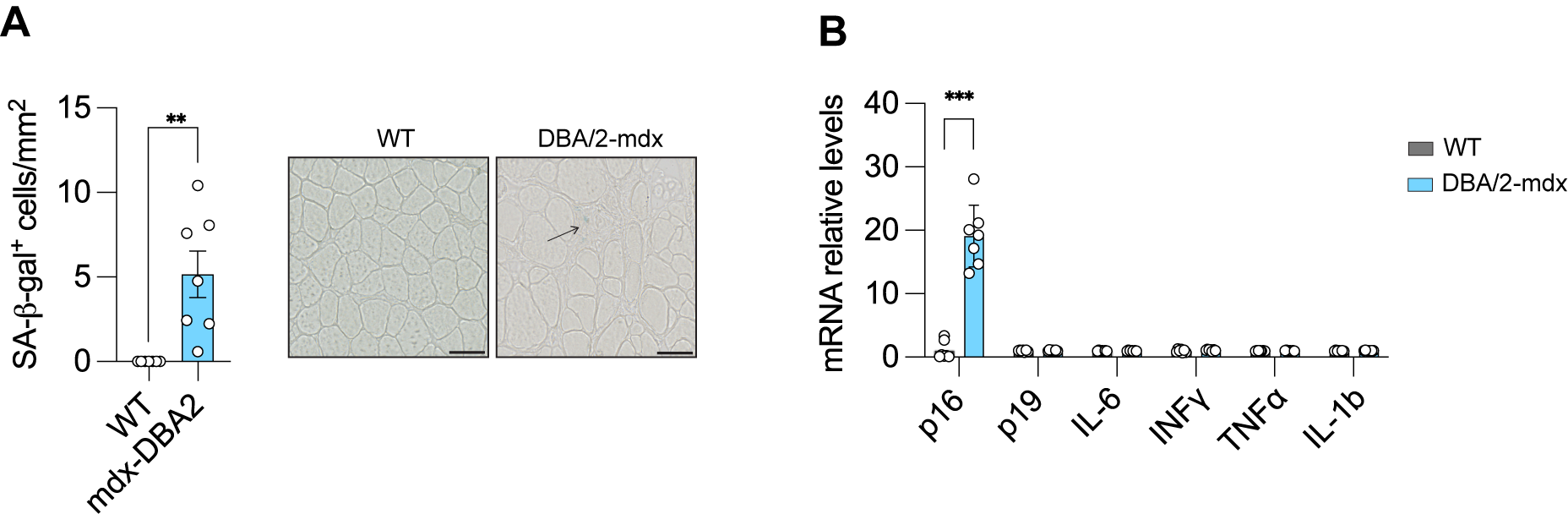

Supplement: Supplementary file 1 — Supplementary Material 1: Supplementary Fig. 1. Senescent cell markers in soleus muscles of WT or DBA/2-mdx young mice. (A) Quantification of SA-β-gal⁺ cells and representative images of young WT and DBA/2-mdx mice. (B) Relative mRNA levels of senescent markers in the soleus of WT and DBA/2-mdx mice at young ages assessed by RT-qPCR. Data are presented as mean ± SEM. The unpaired Student's t-test was used in all comparisons (DBA/2-mdx vs WT). Statistical significance is indicated by *p < 0.05; **p < 0.01; ***p < 0.001; and ****p < 0.0001. Scale bar, 50 µm. [file 13395_2026_426_MOESM1_ESM.tif]

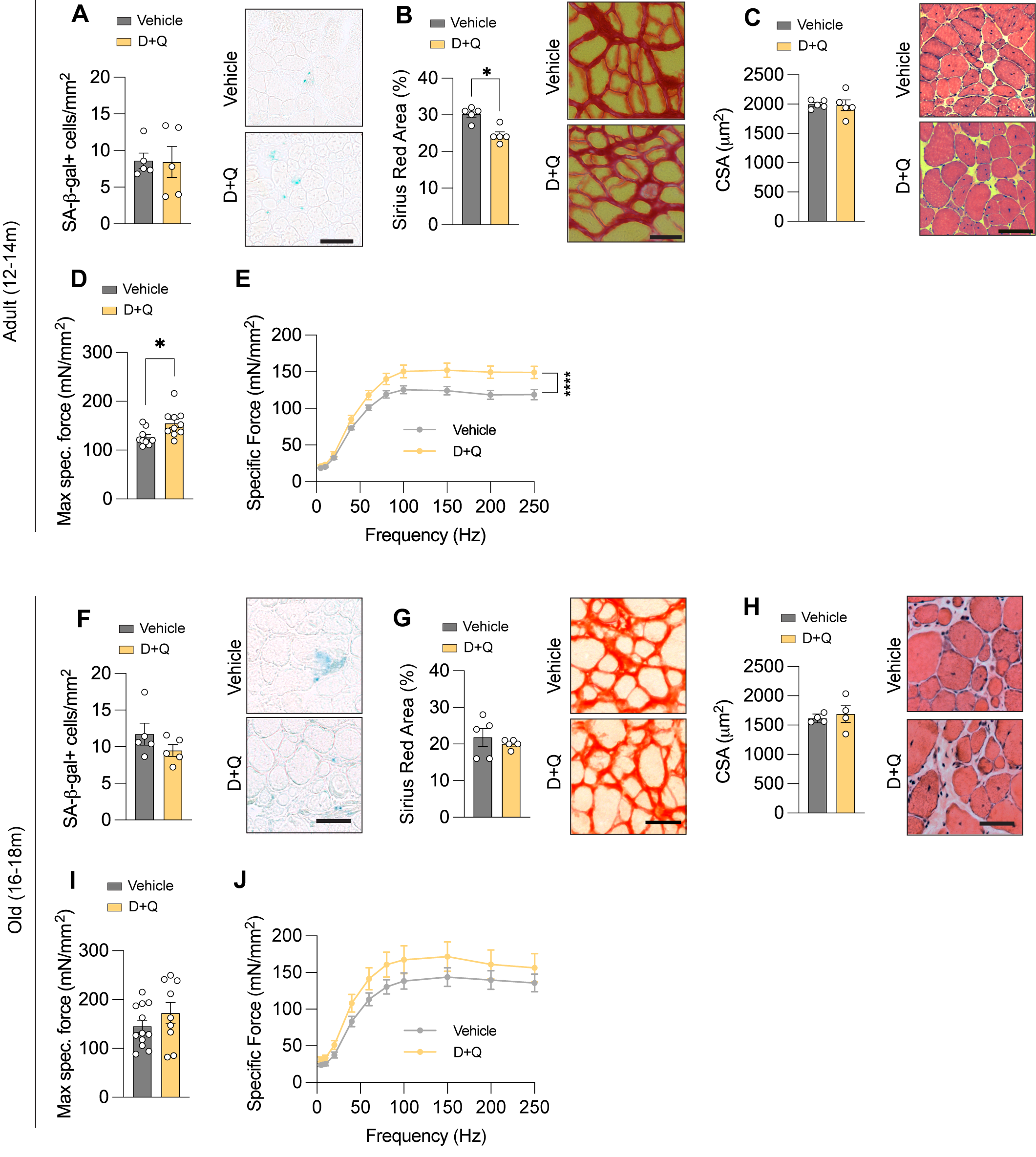

Supplement: Supplementary file 2 — Supplementary Material 2: Supplementary Fig. 2. Effects of senolytic treatment in the skeletal muscle of adult and old dystrophic mice. Adult (12 months; A–E) and old (> 16 months; F–J) DBA/2-mdx mice were treated with vehicle or D+Q twice weekly for 8 weeks. (A, F) Quantification and representative images of SA-β-gal⁺ cells in the TA muscle of DBA/2-mdx mice. (B, G) Quantification of collagen deposition and representative images of Sirius Red staining. (C, H) Mean cross-sectional area (CSA) fibers in the TA of DBA/2-mdx mice based on H&E-stained sections. (D, I) Maximum specific force and (E, J) force–frequency curves of the EDL muscle from vehicle- and D+Q–treated mice. The Mann–Whitney test was used for all comparisons. Two-way ANOVA was used for the force-frequency curve analysis. Statistical significance is indicated by *p < 0.05; **p < 0.01; ***p < 0.001; and ****p < 0.0001. Scale bars, 50 µm. [file 13395_2026_426_MOESM2_ESM.tif]

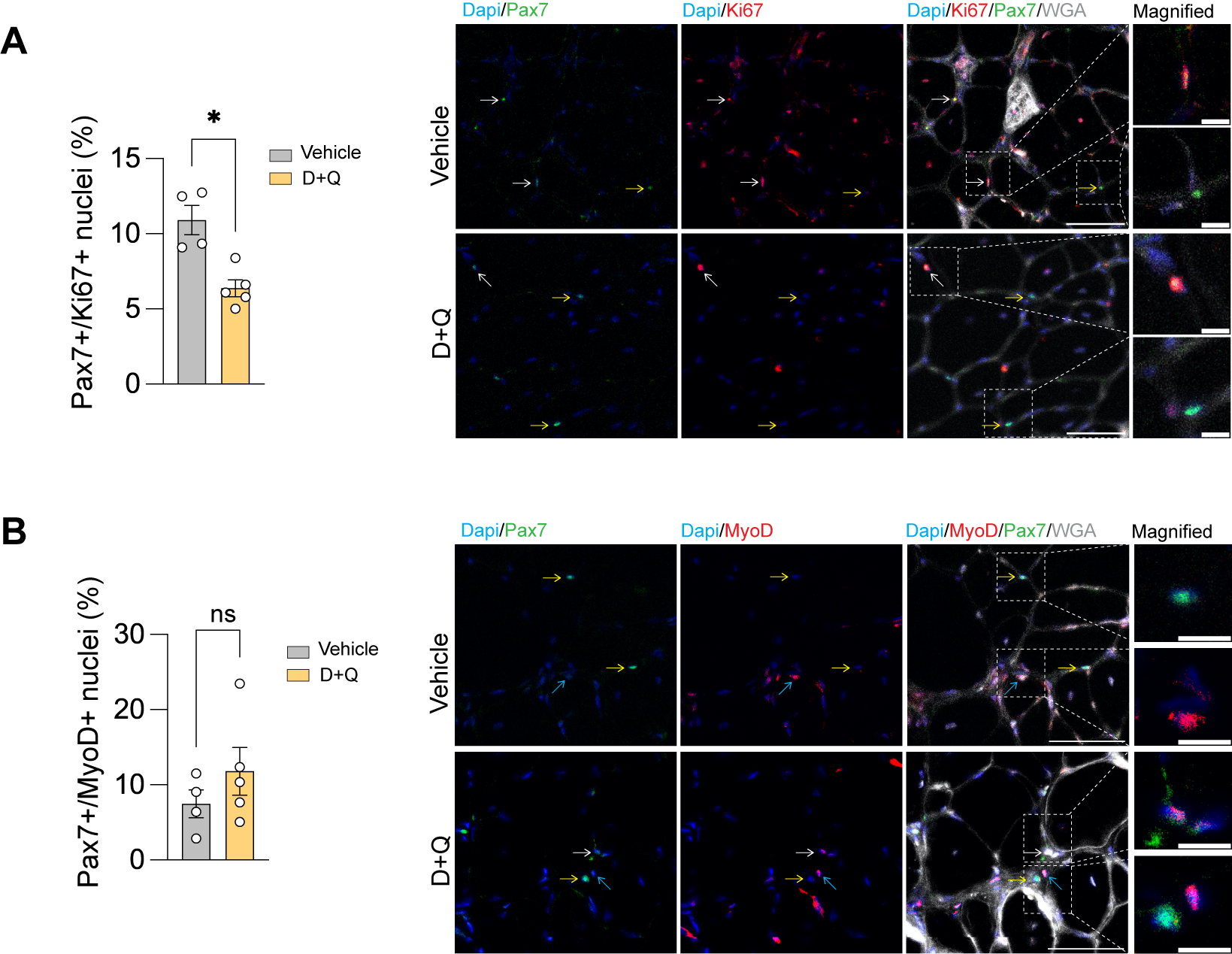

Supplement: Supplementary file 3 — Supplementary Material 3: Supplementary Figure 3. (A) Quantification and representative images of Pax7+/Ki67+ cells in D+Q– and vehicle-treated TAs. (B) Quantification and representative images of Pax7+/MyoD+ cells in D+Q– and vehicle-treated TAs. Yellow arrows show Pax7+/Ki67- nuclei (in A) or Pax7+/MyoD- nuclei (in B); white arrows show Pax7+/Ki67+ nuclei (in A) or Pax7+/MyoD+ nuclei (in B); blue arrows show Pax7-/MyoD+ nuclei. Data are presented as mean ± SEM. The Mann–Whitney test was used for all comparisons. Statistical significance is indicated by *p < 0.05. Scale bars, 50 µm. Scale bars of magnified images, 10 µm. [file 13395_2026_426_MOESM3_ESM.tif]

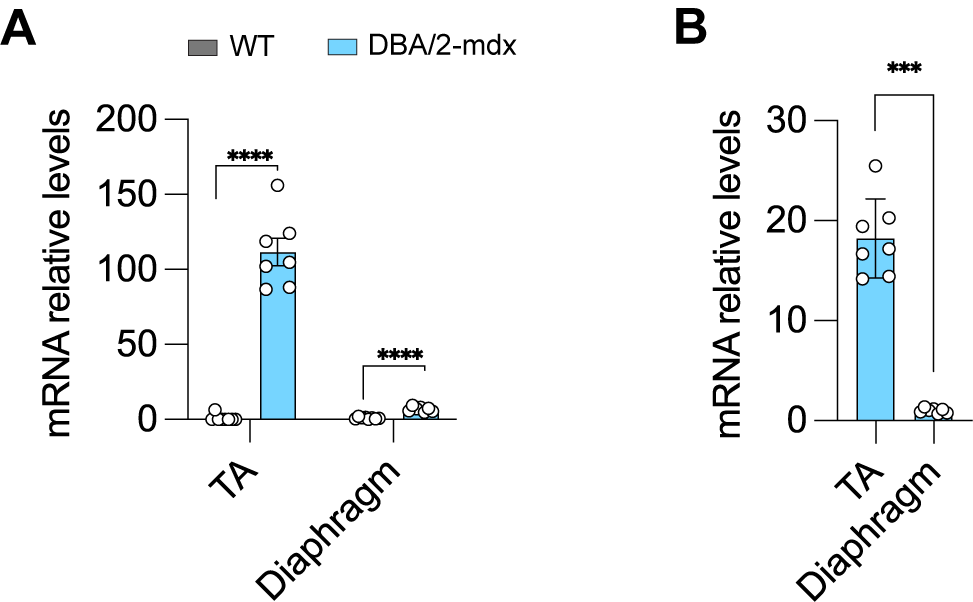

Supplement: Supplementary file 4 — Supplementary Material 4: Supplementary Fig. 4. p16 mRNA levels in the TA and diaphragm of DBA/2-mdx mice. (A) mRNA levels of p16 in TA and diaphragm of DBA/2-mdx mice relative to age-matched WT mice. (B) mRNA levels of p16 in TA relative to the diaphragm of the same DBA/2-mdx mice. Data are presented as mean ± SEM. An unpaired t-test was used for all comparisons (in A, individual comparisons were performed between age-matched WT and DBA/2-mdx mice). Statistical significance is indicated by ***p < 0.001; and ****p < 0.0001. [file 13395_2026_426_MOESM4_ESM.tif]

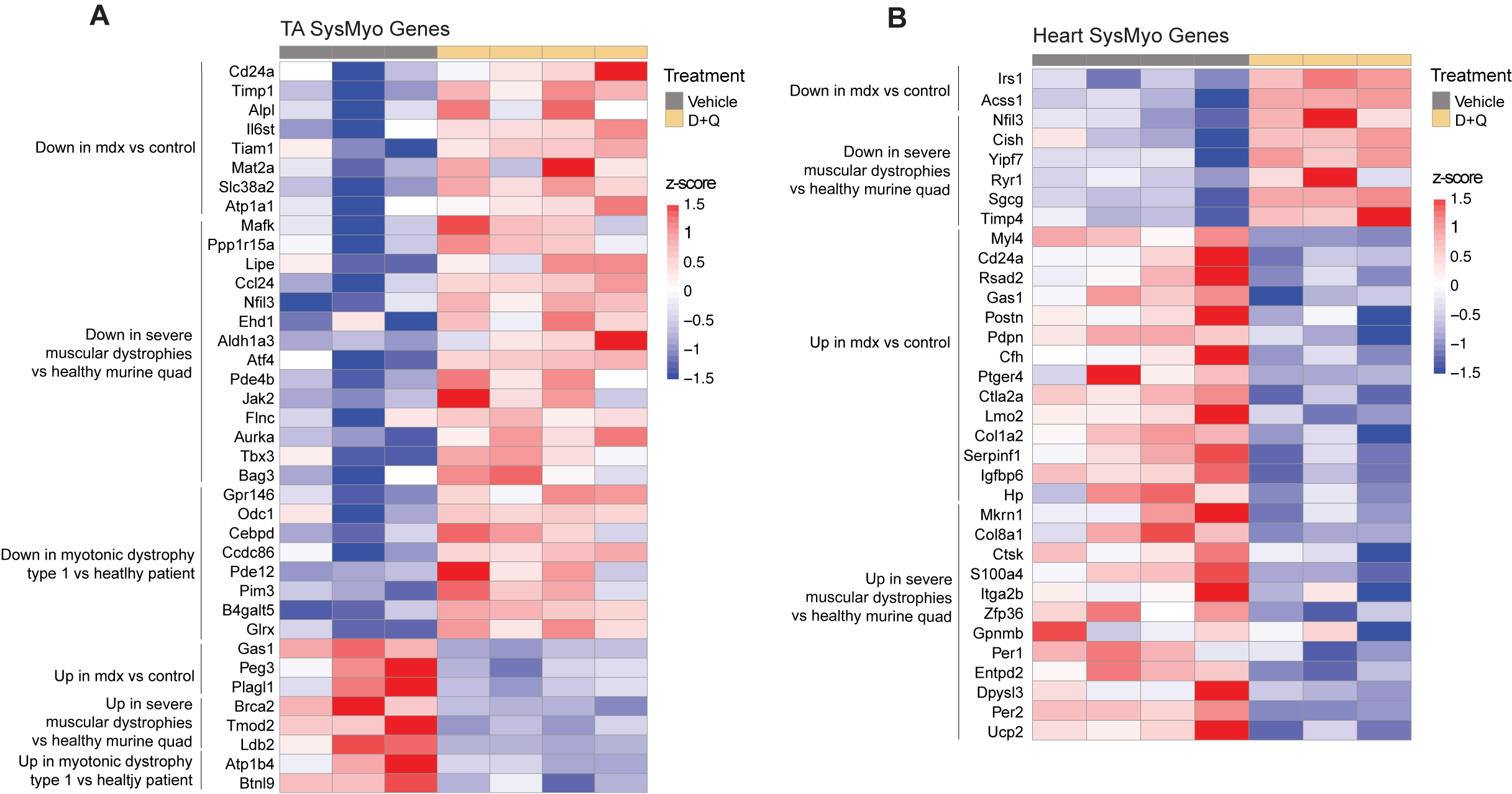

Supplement: Supplementary file 5 — Supplementary Material 5: Supplementary Fig. 5. SysMyo DEGs in TA and heart after D+Q treatment of DBA/2-mdx mice. Heatmaps showing the expression of SysMyo genes upregulated and downregulated in the TA (A) and heart (B) after D+Q treatment of DBA/2-mdx mice. [file 13395_2026_426_MOESM5_ESM.tif]
