# Supplementary material for: Senescence dynamics define therapeutic windows for Duchenne muscular dystrophy in DBA/2-mdx mice
Source: Skelet Muscle. 2026 May 2;16:27. doi: 10.1186/s13395-026-00426-5 (PMC13288804; doi:10.1186/s13395-026-00426-5)
Supplement: Supplementary file 6 — Supplementary Material 6: Supplementary Table 1. DNA Oligos used for RT-qPCR. [file 13395_2026_426_MOESM6_ESM.docx]

**Supplementary table 1. DNA Oligos used for RT-qPCR**

| **Gene** | **Forward Sequence** | **Reverse Sequence** |
| --- | --- | --- |
| Rpl7 | GAAGCTCATCTATGAGAAGGC | AAGACGAAGGAGCTGCAGAAC |
| Cdkn2a (p16) | CATCTGGAGCAGCATGGAGTC | GGGTACGACCGAAAGAGTTCG |
| Cdkn2d (p19) | GCCGCACCGGAATCCT | TTGAGCAGAAGAGCTGCTACGT |
| IL6 | GAGGATACCACTCCCAACAGACC | AAGTGCATCATCGTTGTTCATACA |
| INFγ | GCGTCATTGAATCACACCTG | TGAGCTCATTGAATGCTTGG |
| TNFα | CGCTCTTCTGTCTACTGAACTT | GATGAGAGGGAGGCCATT |
| IL1β | CACAGCAGCACATCAACAAG | GTGCTCATGTCCTCATCCTG |
| Scd4 | ATGCTGGCGGCTCGGATGACT | GGGCTCAATCACTTCAGGGAAG |
| Ppargc1b | CAGCCTCAGTTCCAGAAGTCAG | CACCGAAGTGAGGTGCTTATGC |
