# Supplementary material for: Senescence dynamics define therapeutic windows for Duchenne muscular dystrophy in DBA/2-mdx mice
Source: Skelet Muscle. 2026 May 2;16:27. doi: 10.1186/s13395-026-00426-5 (PMC13288804; doi:10.1186/s13395-026-00426-5)
Supplement: Supplementary file 7 — Supplementary Material 7: Supplementary Table 2. Cardiac function parameters recorded by echocardiography. [file 13395_2026_426_MOESM7_ESM.docx]

**Supplementary Table 2. Cardiac function parameters recorded by echocardiography**

| **Parameter** | **AV±SD Control** | **AV±SD D+Q** | **p-value** |
| --- | --- | --- | --- |
| EF | 74.70 ± 9.58 | 80.75 ± 4.24 | 0.0796 |
| FS | 41.46 ± 8.53 | 46.38 ± 3.37 | 0.1033 |
| SV | 25.61 ± 12.31 | 29.71 ± 5.98 | 0.3488 |
| CO | 10.26 ± 5.21 | 12.54 ± 2.59 | 0.2237 |
| LVAWd | 0.960 ± 0.28 | 0.796 ± 0.16 | 0.2331 |
| LVPWd | 1.251 ± 0.29 | 1.197 ± 0.40 | 0.7240 |
| LVIDd | 2.937 ± 0.62 | 3.099 ± 0.27 | 0.4550 |
| IVSd | 1.107 ± 0.27 | 0.992 ± 0.17 | 0.2729 |
| LVAWs | 1.391 ± 0.26 | 1.452 ± 0.16 | 0.6380 |
| LVPWs | 1.701 ± 0.34 | 1.603 ± 0.39 | 0.5399 |
| LVIDs | 1.538 ± 0.35 | 1.711 ± 0.16 | 0.1732 |
| IVSs | 1.594 ± 0.47 | 1.643 ± 0.24 | 0.7735 |
| Heart rate | 410.6 ± 76.82 | 424.4 ± 54.40 | 0.6470 |
| EDV | 35.51 ± 17.52 | 38.32 ± 8.49 | 0.6480 |
| ESV | 9.901 ± 6.96 | 8.613 ± 2.68 | 0.5887 |
| RWT | 0.896 ± 0.33 | 0.778 ± 0.28 | 0.3774 |
| SI | 2.545 ± 0.30 | 2.444 ± 0.12 | 0.3313 |
| LVm | 113.8 ± 30.32 | 109.2 ± 28.05 | 0.7150 |
| Aortic diameter | 1.342 ± 0.15 | 1.357 ± 0.16 | 0.8374 |
| A peak | 0.465 ± 0.27 | 0.553 ± 0.32 | 0.5218 |
| E peak | 0.553 ± 0.20 | 0.644 ± 0.21 | 0.3440 |
| E/A | 1.455 ± 0.66 | 1.432 ± 0.54 | 0.9363 |
| VTI | 2.249 ± 1.13 | 2.609 ± 1.25 | 0.5100 |

The heart function of DBA/2-mdx mice treated with D+Q from 12 to 16 month of age was assessed by echocardiography at the end of the treatment. Different parameters were evaluated. Abbreviations: A peak, peak late diastolic transmitral flow velocity; CO, cardiac output; E/A, ratio of E peak and A peak; EDV, end-diastolic volume; EF, ejection fraction; E peak, peak early diastolic transmitral flow velocity; ESV, end-systolic volume; FS, fractional shortening; IVSdm, interventricular septal thickness in diastole; IVSs, interventricular septal thickness in systole; LVAWd, left ventricular anterior wall in diastole; LVAWs, left ventricular anterior wall in systole; LVIDd, left ventricular internal diameter in diastole; LVIDs, left ventricular internal diameter in systole; LVm, left ventricular mass; LVPWd, left ventricular posterior wall in diastole; LVPWs, left ventricular posterior wall in systole; RWT, relative wall thickness; SI, sphericity index; SV, stroke volume; VTI, velocity time integral.
